# How is patient‐centred care conceptualized in obstetrical health? comparison of themes from concept analyses in obstetrical health‐ and patient‐centred care

**DOI:** 10.1111/hex.13434

**Published:** 2022-01-13

**Authors:** Kelly Dong, Bismah Jameel, Anna R. Gagliardi

**Affiliations:** ^1^ Department of Medicine University of Toronto Toronto Ontario Canada; ^2^ Toronto General Hospital Research Institute University Health Network Toronto Ontario Canada

**Keywords:** literature review, maternal health, patient‐centred care, patient preferences, person‐centred care, quality of care, women's health

## Abstract

**Background:**

Due to gender inequities that exist for women of childbearing age, there exists a need to deliver care tailored to their needs and preferences. Patient‐centred care (PCC) can be used to meet these needs. This review aims to compare patient care delivery between PCC and obstetrical care. This can help us address how PCC should be delivered to women before, during and after pregnancy versus how it is delivered to patients regardless of sex.

**Methods:**

A review of literature was conducted on MEDLINE, EMBASE, CINAHL and SCOPUS for English PCC and high‐quality perinatal reviews published between 2010 and 2021. The data were analysed using a modified Walker and Avant framework.

**Results:**

A total of 2138 unique studies were identified, with 11 PCC and 9 high‐quality obstetrical care studies included. Common defining features between PCC and obstetrical care include respect and dignity, informed decision‐making, therapeutic alliance, effective communication, social relationships and autonomy. PCC‐specific features were holistic care, empowerment, individualized care, coordinated care and empathy. Unique high‐quality obstetrical care themes included continuity of care, privacy and confidentiality, provider education and status, physical environment and equitable maternal care.

**Conclusions:**

There are shared defining attributes between PCC and obstetrical care, including respect and dignity, informed decision‐making, the therapeutic alliance, effective communication, social relationships and autonomy. However, there remain unique defining attributes for high‐quality obstetrical care and PCC. This highlights the need for a unique approach to obstetrical care. More research on care for different physiological conditions in women is needed to address patient care that addresses different parts of the lifespan and develop frameworks that can influence health policy, patient care and health system evaluation.

**Patient or Public Contribution:**

This study was one part of a larger, multicomponent study of how to implement PCC for women across the lifespan. While we did not specifically consult or involve women in this dual concept analysis, our larger study (content analysis of clinical guidelines and government policies, qualitative interviews with women and clinicians, Delphi study to prioritize consensus recommendations for achieving PCC for women) was guided by the experiences and input of a 50+ women advisory panel.

## 1 INTRODUCTION

Patient‐centred care (PCC) was defined by the Institute of Medicine as care that establishes a partnership among practitioners, patients and their families to ensure that providers and systems deliver care that is attentive to the needs, values and preferences of patients.^1^ Since then, considerable research has expanded our understanding of PCC and how to achieve it. For example, a scoping review of 19 studies published from 1994 to 2011 identified 25 unique frameworks or models of PCC^2^ and several validated instruments with which to measure PCC.^3^ Common elements of PCC include effective communication, partnership and health promotion.^2^, ^3^ Another review of 28 reviews published between 2011 and 2017 identified a variety of informational, educational and supportive interventions that can be used to achieve PCC targeted at patients, family members or providers.^4^ PCC is now widely recognized as a fundamental element of high‐quality health care because it has been associated with numerous beneficial outcomes for patients (i.e., increased knowledge, skill, satisfaction, quality of life; decreased admissions, readmissions and length of hospital stay), family members (increased satisfaction; decreased stress and anxiety) and provider (improved job satisfaction, confidence and quality of care; reduced stress and burnout) outcomes across multiple settings, including primary, emergency, acute and intensive care.^4^, ^5^, ^6^

Still, many patients do not receive or experience PCC. For example, a national survey in the United States showed that, among 2718 responding adults aged 40 years or older with 10 common medical conditions, there was considerable variation in perceived PCC among patients including involvement in discussing treatment options and making decisions.^7^ Suboptimal PCC was reported by half of 1794 American cancer survivors responding in 2013 to a national survey.^8^ In 2016, a Commonwealth Fund national survey revealed that fewer women reported patient‐centred communication with their provider compared with the general population.^9^ Women continue to experience gendered inequities in access to and the quality of care in both developed and less developed countries,^10^, ^11^ leading to national and international appeals over several decades to improve PCC for women.^12^, ^13^, ^14^, ^15^, ^16^ Despite evidence of inequities and appeals to improve PCC for women, little research has identified how to promote and support PCC for women. We conducted a theoretical rapid review to describe how PCC was studied among women affected by depression or cardiovascular disease, conditions with known gendered inequities.^17^ Our review identified a few studies of PCC among women, and those studies failed to fully conceptualize or describe PCC. We subsequently explored women's and clinicians' views about what constitutes PCC,^18^ and generated recommendations by which to achieve PCC for women.^19^

PCC could address gendered inequities by engaging women in their care and tailoring care to their needs and values. Hence, further research is needed to explore how to foster PCC for women with different conditions or healthcare issues. Giving birth is one of the most common reasons for inpatient hospitalisation, and the cost of inpatient delivery is increasing over time despite declining pregnancy rates.^20^ Quality of care during labour and birth affects maternal and child morbidity and mortality, and is a concern worldwide.^21^ Factors such as lack of coordinated care among providers, fragmentation of care and substandard care also negatively influence patient‐centred obstetric care.^22^ A systematic review of 47 studies on person‐centred interventions in delivery facilities found that interventions aimed to improve autonomy, supportive care, social support, health facility environment and dignity, but the person‐centred objectives did not match the PCC or clinical outcomes measured.^23^ The authors emphasized this lack of theoretical coherence between aims and intervention design, given that interventions to improve quality of care are more successful when selected and tailored according to preidentified barriers and theory, which may lead to more thorough measurement and evaluation of PCC in maternity care.^24^ Hence, there is a need to more thoroughly conceptualize PCC in maternal care to inform the development of interventions that improve the quality of maternal care and of measures to assess their impact.

Primary research in maternity care has focused on the experiences of women in maternity care,^25^, ^26^ goals of maternity care,^27^ interventions to improve quality of maternity care^28^ and outcomes of high‐quality maternity care.^29^ However, few reviews have synthesized these elements, and no prior reviews mapped the domains of high‐quality maternity care to PCC domains or a PCC framework. The purpose of this study was to compare the concepts of PCC with concepts of high‐quality inpatient obstetric care in published conceptual reviews. This would identify common elements and potentially PCC elements unique to the maternal care context by which to plan and improve obstetrical care for women giving birth as inpatients. This knowledge could be used by women's health researchers, and also by clinicians, and healthcare managers and policy‐makers to inform the planning, delivery and improvement of healthcare services for women.

## 2 MATERIALS AND METHODS

### 2.1 Approach

The main purpose of this review is to compare and contrast the elements of PCC and high‐quality obstetrical care found in the literature. To do so, we conducted a concept analysis, which is a ‘process of determining the likeness and unlikeness between concepts’^30^ that has been used by others to compare models of quality of life^31^ and patient participation.^32^ More specifically, we used the Walker and Avant^30^ concept analysis approach. Other approaches such as the Rogers' evolutionary concept analysis or Haase's simultaneous concept analysis built upon this model, but the Walker and Avant model remains the approach most widely used.^33^ The approach includes choosing a concept, determining the purpose of analysis, identifying all uses of the concept, defining attributes, identifying antecedents and consequences and defining empirical referents. This provides a comprehensive understanding of each topic independent of each other as well as a comparison of the defining attributes, antecedents, consequences and empirical referents that are shared between these two topics. This was completed by conducting a review of literature between 2010 and 2021 for reviews that examine patient care in PCC and obstetrical care. The two primary objectives of this review are to (1) gain an understanding of how PCC and obstetrical care has been conceptualized since 2010 and (2) to compare the characteristics of patient care between these two concepts. This will provide a foundation for PCC for women based on the identified values and preferences of female patients in the birthing process.

### 2.2 Eligibility criteria

Detailed inclusion and exclusion criteria (File S1) were based on persons/participants, issue/intervention, comparisons and outcomes.^34^ In brief, for the PCC concept analysis, the persons or participants were any patients aged 18+ or healthcare professionals in any primary, secondary or tertiary setting of care. The interventions were reviews that examine or describe elements and processes that constitute person‐centred care. The comparisons were what participants view as PCC or PCC barriers, or assess if PCC was delivered, or evaluate PCC outcomes after an intervention, before and after an intervention or compared between interventions. The outcomes were views, beliefs or preferences, enablers, barrier or challenges, interventions that promote or support PCC and impacts of PCC. Reviews were excluded if they focused on a specific population or clinical situation (e.g., palliative care, paediatric population, emergency).

The high‐quality obstetrical care concept analysis included patients 18+ receiving obstetrical or reproductive care during labour and delivery or the perinatal period or healthcare professionals who provide obstetrical care. The intervention was high‐quality perinatal care. Comparisons were also performed on participant views, high‐quality obstetrical care delivery, evaluation of perinatal outcomes after an intervention, before and after an intervention or compared between interventions. The outcomes were views, beliefs or preferences, enablers, barriers or challenges, interventions that promote or support high‐quality obstetrical care and impacts of high‐quality care. Reasons for exclusion were if the reviews focused on a specific aspect of obstetrical care outside the immediate labour and delivery experience (antenatal care, breastfeeding, ectopic pregnancies or termination).

### 2.3 Search strategy

B. J. and K. D. searched MEDLINE, EMBASE, CINAHL and SCOPUS databases on 29 March 2021. This included English syntheses of the literature between the years of 2010 and 2021 for PCC and perinatal care separately. Our preliminary searching revealed several existing reviews on the topics of PCC and maternal care, so rather than including both primary studies and reviews, potentially resulting in overlapping studies, we chose to include only reviews, as they represent the totality of published information on a given topic (see File S2 for the search strategy in MEDLINE). The search terms for PCC studies included patient‐, client‐, family‐ and woman‐centred care. Both American and British spellings were used and variations of search terms with or without hyphens. The systematic review for obstetrical studies included terms such as obstetric, birth, postnatal, perinatal, labour and delivery to search for the obstetric reviews that examine the labour process. This was combined with terms for healthcare quality, quality improvement, patient satisfaction, quality assurance, quality indicators, programme evaluation and provider–patient relations. These results were limited to English‐language reviews that are reviews of literature. A total of 2136 records were exported from all databases once duplicates were removed.

### 2.4 Screening

B. J. and K. D. screened the titles and abstracts for articles that fulfilled the eligibility criteria. Subsequently, B. J. and K. D. conducted a more refined screening of full‐text articles that were relevant to the PCC or obstetrical care. Articles were excluded if they were not reviews, focused on provider perspectives. Studies were also excluded if they discussed concepts such as patient‐reported outcome (PRO) measures, PROs, patient navigation, patient activation or specific interventions or tools. Specific populations of uses or populations were also excluded, such as end‐of‐life care, residential or long‐term care, palliative care, emergency medicine, paediatric populations or any other focused group. Studies that were clinically focused on the illness rather than the care experience were not included. Articles that mentioned PCC in the background or conclusion without explicitly focusing on PCC were also excluded.

### 2.5 Data extraction

K. D. and B. J. conducted a pilot data extraction that was reviewed by A. R. G. to ensure consistency; author, year, country, study design, findings and definitions of PCC or high‐quality obstetrical care. The study design included the type of review, data range of the included articles and number of articles included. Studies were not appraised for quality.

### 2.6 Data analysis

B. J. and K. D. first extracted direct quotations from the studies with their primary results. The data were analysed by K. D. based on the Walker and Avant domains, which were defining attributes, antecedents, consequences and empirical referents. Unique themes were identified if they appeared across multiple studies or were significant themes identified in the included studies. The identified defining attributes, antecedents, consequences and empirical referents were reviewed with A. R. G. recursively before consolidating a final list. The data were analysed first within PCC and obstetrical care independently before comparing the overlap and unique elements of the two concepts. The identified themes remained close to the wording that was used by the studies. Themes that were very similar in meaning, for instance, ‘unique to person’ and ‘individualized care’ or ‘autonomy’ and ‘ownership and control’, were combined into one term.

## 3 RESULTS

### 3.1 Search results

**Figure 1:**
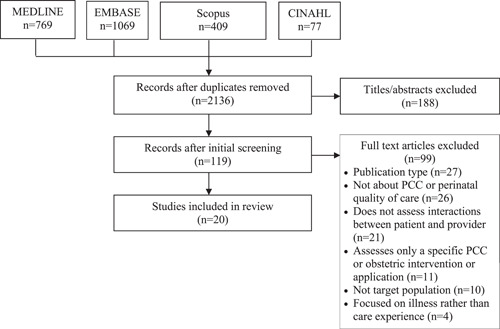
PRISMA diagram. PCC, patient‐centred care; PRISMA, Preferred Reporting Items for Systematic Reviews and Meta‐Analyses

The search yielded 2324 studies, and 188 duplicates were removed (see Figure 1). A total of 119 full‐text articles were screened by B. J. and K. D. Of these, 99 were excluded due to publication type (*n* = 27), focus not being on PCC or perinatal quality of care (*n* = 26), not assessing interactions between patient and provider (*n* = 21), assessed only a specific PCC or obstetrical intervention or application (*n* = 11), did not focus on the target population (*n* = 10) and focused on illness rather than care experience (*n* = 4). Ultimately, 20 studies were included, which consisted of 11 PCC and 9 high‐quality obstetrical care reviews (refer to Table 1 for the characteristics of the included studies).

**Table 1:** Characteristics of included PCC and obstetrical reviews

Study	Objective	Review details	Definitions	Defining attributes	Antecedents	Consequences	Empirical references
PCC reviews							
Najafizada 2021 Canada	Identify and synthesize models of patient‐centred care in Canada and compare them with the normative models described in the literature	Scoping review and environmental scan 2010–March 2019 19 articles	Definition of PCC: (1) Considering patients' needs, wants, perspectives and individual experiences; (2) offering patients opportunities to provide input into and participate in their care; and (3) enhancing partnership and understanding in the patient–physician relationship	Respect and dignity Autonomy Individualized care	Organisational capacity	Improved quality of care Provider satisfaction Improved trust	None
Jaensch 2019 Australia	To understand the domains of agreement and disagreement, related to person‐centred care, between the patient and healthcare professional during a shared episode of care	Systematic review No restriction—February 2019 15 articles	NR (not reported)	Communication Respect and dignity Empathy Individualized care	Organisational capacity Provider qualities	Patient engagement Patient satisfaction Improved trust Treatment adherence	None
Langberg 2019 Denmark	Overview of patient‐centred and updated definition of the concept	Systematic review January 2000–June 2017 80 articles	Definition of PCC: Understanding the patient's situation, developing the doctor–patient relationship and managing coordination of care in the organisational framework of the healthcare system	Holistic care Individualized care Coordinated care Therapeutic alliance Autonomy	Patient factors Organisational capacity Provider qualities	Empowerment Improved quality of care Reduced healthcare resource utilisation	NR (not reported)
Eklund 2019 Sweden	To provide a synthesis of already synthesized literature on person‐centred care and patient‐centred care to identify similarities between the two concepts	Meta‐review January 2000–March 2017 21 articles	NR (not reported)	Respect and dignity Relationships Therapeutic alliance Empathy Communication Informed decision‐making Holistic care Individualized care Coordinated care	NR (not reported)	Improved quality of care Patient satisfaction	NR (not reported)
Coyne 2018 Ireland	To describe and clarify family‐centred care, person‐centred care and child‐centred care	Concept Synthesis January 2012–December 2016 31 articles	Definition of PCC: A holistic approach to delivering care that is respectful and individualized, allowing negotiation of care and offering choice through a therapeutic relationship where persons are empowered to be involved in health decisions at whatever level is desired by that individual who is receiving the care	Holistic care Individualized care Respect and dignity Empathy Autonomy	Patient factors Provider qualities Shared governance	Improved quality of care Patient satisfaction Improved health outcomes	NR (not reported)
Waters and Buchanan 2017 Australia	Identify the uses, definitions and characteristics of the term ‘person‐centred’ in the ageing, mental health and disability literature	Scoping review and thematic analysis 1995–2015 504 articles	NR (not reported)	Individualized care Informed decision‐making Autonomy Relationships Therapeutic alliance Empathy	NR (not reported)	Improved trust Patient engagement Provider satisfaction	NR (not reported)
Castro 2016 Belgium	Clarify the meaning of the overlapping concepts of patient empowerment, patient participation and patient‐centredness by highlighting their interrelationship and distinguishing their antecedents, attributes, consequences and empirical referents and this is the aim of improving understanding between different groups of healthcare professionals in hospital care. A second goal is to suggest a definition as well as a process model for these three concepts	Concept analysis 2006–2016 103 articles, 36 articles used for patient‐centredness	NR (not reported)	Holistic care Respect and dignity Relationships Individualized care Empathy	Clinician–patient communication Organisational capacity Provider qualities	Improved health outcomes Improved quality of care Patient satisfaction Treatment adherence Reduced healthcare resource utilisation Patient knowledge and health literacy	Individualized care: Individualized Care Scale Empathy: The Consultation and Relational Empathy Scale Person‐centred climate: Person‐centred Climate Questionnaire Patient Version Patient‐centred care and patient–caregiver relationship: Client‐Centred Care Questionnaire Quality of the therapeutic alliance: Kim Alliance Scale A patient–doctor relationship. A Patient–Doctor Relationship Questionnaire Interpersonal trust Stanford Trust in Physician Scale
Lor 2016 USA	To explicate and compare four conceptual care models: person‐, patient‐, family‐centred, and culturally competent care	Comparative concept analysis 2009–2013 32 articles	NR (not reported)	Therapeutic alliance Communication Respect and dignity Holistic care Individualized care Relationships Empowerment	Clinician–patient communication Organisational capacity Patient factors Systemic factors	Improved health outcomes Patient satisfaction Improved trust	NR (not reported)
Scholl 2014 Germany	Identify the different dimensions of patient‐centredness described in the literature and to propose an integrative model of patient‐centredness based on these results	Systematic review and concept analysis from database inception—January 2012 417 articles	NR (not reported)	Therapeutic alliance Individualized care Holistic care Informed decision‐making Communication Relationships Empowerment	Clinician–patient communication Provider qualities Systematic factors Organisational capacity	NR (not reported)	NR (not reported)
Lusk 2013 USA	The purpose of this article is to describe a concept analysis using Walker and Avant's method. Multiple terms inherent to PCC are explored	Concept analysis Included studies from 2001 to 2010 24 articles	Definition of PCC: The provision of care incorporating contextual elements and including the attributes of encouraging patient autonomy, the caring attitude of the nurse and individualizing patient care by the nurse	Autonomy Informed decision‐making Empathy Respect and dignity Therapeutic alliance Individualized care	Need for healthcare intervention Patient factors	Patient satisfaction Improved quality of care Improved health outcomes	Health Care Climate Questionnaire Schmidt perception of the Nursing Care Survey
Morgan and Yoder 2012 USA	This article uses Walker and Avant's method of concept analysis as a framework to analyse PCC	Concept analysis No date restrictions 50 articles	NR (not reported)	Holistic care Individualized care Empowerment Respect and dignity	Provider qualities Organisational capacity Shared governance	Improved quality of care Patient satisfaction Improved health outcomes	Person‐Centred Climate Questionnaire Individualized Care Scale Patient‐Centred Inpatient Scale Patient Satisfaction with Nursing Care Quality Questionnaire Short Form‐36 Functional Independence Measurement
Obstetrical reviews							
Hulsbergen 2020 Tanzania	Identify different aspects of quality of care and respectful care in relation to maternal healthcare and the influence of these aspects of care on the uptake of skilled birth attendance in Tanzania	Narrative review 2009–2019 Unknown number of studies	Definition of respectful maternal care: Care organized for and provided to all women in a manner that maintains their dignity, privacy and confidentiality, ensures freedom from harm and mistreatment and enables informed choice and continuous support during labour and childbirth	Autonomy Informed decision‐making Privacy and confidentiality Equitable maternal care Provider education and status	Provider qualities Organisational capacity Systemic factors	Patient satisfaction Trust Patient knowledge and health literacy	NR (not reported)
Megregian 2020 USA	Identify impact of shared decision‐making	Scoping review 2000–2019 9 articles	Definition of patient‐centred Care: Approach to health care that uses a holistic framework to address a person's health and well‐being and has been linked to improved patient satisfaction, provider–patient communication and health outcomes Definition of shared decision‐making: Collaborative process in which a healthcare provider and patient engage with one another to make healthcare decisions, using respectful communication and basing their decisions on the best available evidence and the patient's preferences, values and goals	Informed decision‐making	Patient factors	Self‐efficacy Patient satisfaction Patient knowledge and health literacy	Perinatal outcomes (e.g., VBAC vs. planned repeat caesarean birth) Decisional regret scores Shared Decision‐Making Questionnaire (SDM‐Q‐9)
Ansari 2020 India	Identify forms of disrespectful maternity care, determinants and pooled prevalence during childbirth in India	Systematic review 2016–2019 7 articles	NR (not reported)	Privacy and confidentiality Informed decision‐making	Organisational capacity Patient factors	Respectful intrapartum care	Prevalence of ill‐treatment (nonconsent, verbal abuse, threats, physical abuse and discrimination)
Fair 2020 Europe	Provide evidence on migrant women's experiences of pregnancy, childbirth and maternity care in their destination European country	Systematic review 2007–2017 51 articles	Definition of quality maternity care: Trusting relationship between women and HCPs, which empowered women to feel confident and prepared for childbirth, even overcoming a lack of social networks or support	Informed decision‐making Communication Therapeutic alliance Respect and dignity	Systemic Factors Organisational capacity	Patient satisfaction Perinatal outcomes Respectful intrapartum care	NR (not reported)
Coates 2019 United Kingdom	To explore and synthesize evidence of women's experiences of induction of labour (IoL)	Qualitative systematic review and thematic synthesis 2010–2018 11 articles		Informed decision‐making Autonomy Social relationships Physical environment	Mutual participation and communication	Patient satisfaction	NR (not reported)
Akuamoah‐Boateng 2018 United Kingdom	Aimed to explore women's experiences and perceptions of IoL for uncomplicated post‐term pregnancy in a bid to provide a woman‐centred approach to the care of women with uncomplicated postterm pregnancy	Qualitative systematic review No date range given 5 articles	Definition of woman‐centred care: Seeks to provide each individual woman with the appropriate information in a manner that promotes participation and enhanced informed decision‐making. It also places particular emphasis on each woman's particular need and specific situation	Informed decision‐making	Patient factors Provider qualities	Attitudes about future pregnancies Relationship with their babies in the future	NR (not reported)
Shakibazadeh 2017 Iran	To describe how respectful maternity care (RMC) is conceptualized in healthcare settings and facilities across the world	Qualitative evidence synthesis No date range 67 articles	Definition of respectful maternity care: An approach to care that emphasizes the fundamental rights of women, newborns and families, and that promotes equitable access to evidence‐based care while recognizing the unique needs and preferences of both women and newborns	Privacy and confidentiality Respect and dignity Informed decision‐making Continuity of care Physical environment Equitable maternal care	Provider qualities	Perinatal outcomes	NR (not reported)
Bradley 2016 United Kingdom	Review of the growing literature on women's experiences of facility‐based delivery in sub‐Saharan Africa to examine the drivers of disrespectful intrapartum care	Systematic review and meta‐synthesis 1990–2015 25 articles	NR (not reported)	Autonomy Provider education and status	Systemic Factors Provider qualities Organisational capacity	Respectful intrapartum care	NR (not reported)
Maputle 2013 South Africa	To conduct a concept analysis of woman‐centred care in the context of childbirth	Concept analysis Date range not specified 31 articles	Woman‐centred care: A holistic approach, with professionals and family working collaboratively towards a common outcome	Informed decision‐making Autonomy Social Relationships	Mutual participation and communication Provider qualities	Self‐efficacy Trust	NR (not reported)

Abbreviations: HCP, healthcare patients; PCC, patient‐centred care; VBAC, vaginal birth after caesarean section.

### 3.2 Study characteristics

A total of 11 PCC and 9 obstetrical care reviews were included.^35^, ^36^, ^37^, ^38^, ^39^, ^40^, ^41^, ^42^, ^43^, ^44^, ^45^, ^46^, ^47^, ^48^, ^49^, ^50^, ^51^, ^52^, ^53^, ^54^ These were from the United States of America (*n* = 4), United Kingdom (*n* = 3) and Australia, (*n* = 2), and one each from Canada, Denmark, Sweden, Ireland, Belgium, German, Tanzania, India, Europe, Iran and South Africa. Studies were published from 2012 to 2021. Review types included concept analysis (*n* = 7), systematic review (*n* = 9), scoping review (*n* = 3), narrative review (*n* = 1) and qualitative evidence synthesis (n = 1).

Of the nine high‐quality perinatal care reviews, six offered definitions related to respectful maternity care or woman‐centred care. Only 4 of the 11 PCC reviews provided definitions of PCC.

### 3.3 Defining attributes

**Figure 2:**
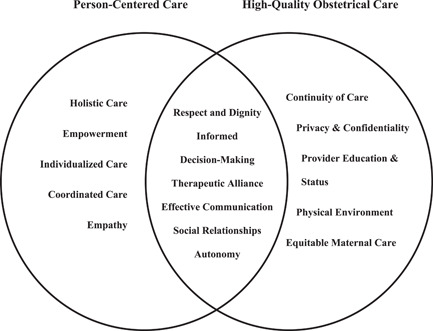
Venn diagram of defining attributes between PCC and high‐quality obstetrical care reviews. PCC, patient‐centred care

High‐quality obstetrical care and PCC shared several common defining features including *respect and dignity*, *informed decision‐making*, *therapeutic alliance*, *effective communication*, *consideration of social relationships* and *patient autonomy* (see Figure 2). Definitions of identified defining attributes were derived from the definitions used in the included studies and can be found in File S3. *Respect and dignity* was a theme in 73% of the PCC studies and 22% of the obstetrical care studies. It was generally defined as affirming the patient choices and perspectives, and in obstetrical care, this definition also extended to freedom from harm and mistreatment. *Informed decision‐making* was present in 36% of PCC studies, but was a much more prominent theme of high‐quality obstetrical care, where 89% of the studies included it as a feature of obstetrical care. The *therapeutic alliance* between the patient and provider was discussed in 55% of PCC and 11% of obstetrical care studies. Effective communication was a defining feature in 36% of PCC and 11% of obstetrical care studies. *Social relationships* were especially important in obstetrical care, where the presence of family or a labour companion influenced their experience of labour. This was in 45% of PCC and 22% of obstetrical care studies. *Autonomy* was a prevalent theme in both PCC and obstetrical care studies, with discussion in 45% and 44%, respectively.

Several PCC‐specific themes were identified, including *holistic care*, *empowerment*, *individualized care*, *coordinated care* and *empathy*. *Individualized care*, which tailored care to the individual perspectives, needs, values and beliefs of the patient, was present in all PCC studies. *Holistic care* was present in 64%, *empowerment* in 27%, *coordinated care* in 11% and *empathy* in 55% of the PCC studies (see Table 2).

**Table 2:** Defining features of the included PCC studies

Study	Respect & dignity	Informed decision‐making	Therapeutic alliance	Effective communication	Social relationships	Autonomy	Individualized care	Empathy	Coordinated care	Empowerment	Holistic care	Number of domains
Najafizada 2021 Canada	x					x	x					3
Jaensch 2019 Australia	x			x			x	x				4
Langberg 2019 Denmark			x			x	x		x		x	5
Eklund 2019 Sweden	x	x	x	x	x		x	x	x		x	9
Coyne 2018 Ireland	x					x	x	x			x	5
Waters and Buchanan 2017 Australia		x	x		x	x	x	x				6
Castro 2016 Belgium	x				x		x	x			x	4
Lor 2016 USA	x		x	x	x		x			x	x	7
Scholl 2014 Germany		x	x	x	x		x			x	x	7
Lusk 2013 USA	x	x	x			x	x	x				6
Morgan and Yoder 2012 USA	x						x			x	x	4
Frequency	8	4	6	4	5	5	11	6	2	3	7	

Abbreviation: PCC, patient‐centred care.

Themes that were unique to high‐quality obstetrical care included *continuity of care, privacy and confidentiality, provider education and status, physical environment* and *equitable maternal care*. The prevalence of these themes was 11% for *continuity of care*, 33% for *privacy and confidentiality*, 22% for *provider education and status*, 22% for *physical environment* and 22% for *equitable maternal care* (see Table 3). *Provider education and status* referred to the education and training that health providers receive and the perception of their own role and status. Studies discussed the *physical environment* as having access to hygienic facilities, quiet and private spaces and adequate space for the labour and delivery process. *Equitable maternal care* was defined as the availability of services to all pregnant women regardless of race, religion, ethnicity or cultural background.

**Table 3:** Defining features of the included obstetrical care studies

Study	Respect & dignity	Informed decision‐making	Therapeutic alliance	Effective communication	Social relationships	Autonomy	Privacy & confidentiality	Continuity of care	Provider education & status	Physical environment	Equitable maternal care	Number of domains
Hulsbergen 2020 Tanzania		x				x	x		x		x	5
Megregian 2020 USA		x										1
Ansari 2020 India		x					x					2
Fair 2020 Europe	x	x	x	x								4
Coates 2019 United Kingdom		x			x	x				x		4
Akuamoah‐Boateng 2018 United Kingdom		x										1
Shakibazadeh 2017 Iran	x	x					x	x		x	x	6
Bradley 2016 United Kingdom						x			x			2
Maputle 2013 South Africa		x			x	x						3
Frequency	2	8	1	1	2	4	3	1	2	2	2	

On average, each PCC study incorporated 5.45 themes (median of 5; range: 3–9). Each obstetrical study incorporated 3.11 themes (median of 3; range: 1–6).

### 3.4 Antecedents and consequences

Antecedents were generally broken down into patient factors, provider qualities, organisational capacity, systematic factors and mutual participation and communication for both PCC and obstetrical studies. File S3 contains the specific antecedents identified in the literature under each category.

In PCC studies, antecedents also included the need for healthcare intervention and shared governance. Patient factors referred to the capacity to engage in decision‐making. Provider qualities ranged from vision and commitment, leadership, personal qualities of the provider, interdisciplinary teamwork to knowledge and ability. Organisational capacity included the physical environment, feedback, access to resources and time and a culture that respects values and choices. Systematic factors, which were present in 18% of the studies, include health disparities and access to care.

The antecedents identified throughout the literature for obstetrical care included patient factors such as their choice predisposition, comfort in expressing preferences, adequate information provision, previous experiences of labour and family/partner involvement. Similar to the obstetrical studies, organisation capacity referred to the availability of resources, health infrastructure, health financing, physical environment that allowed for privacy, care guidelines and continuum of care. Evidence‐based care, quality communication systems, social stability, and cultural sensitivity were systemic factors.

Common consequences between the PCC and obstetrical studies include patient satisfaction, knowledge and health literacy, improved health outcomes and improved trust. Patient satisfaction was a consequence in 64% of the PCC studies and 44% of the obstetrical studies. Improved health outcomes were reported in 45% and 22% of PCC and obstetrical studies, respectively. In PCC studies, improved trust was identified in 36% of the PCC studies and in obstetrical care, improved trust was identified in 22% of the studies. Knowledge and health literacy was a consequence in 9% of PCC studies and 22% of obstetrical care studies. PCC‐specific consequences include improved quality of care, provider satisfaction, patient engagement, treatment adherence, empowerment and reduced healthcare resource utilisation. Improved quality of care was commonly identified in 64% of the PCC studies. Consequences that are unique to high‐quality obstetrical care include respectful intrapartum care, self‐efficacy, attitudes about future pregnancies and future relationship with the child.

### 3.5 Empirical referents

Empirical referents were not commonly identified in PCC or obstetrical care literature. Three PCC reviews listed empirical referents. Tools that were listed to evaluate different domains of PCC include The Consultation and Relational Empathy Scale, the Client‐Centred Care Questionnaire, the Kim Alliance Scale, A Patient–Doctor Relationship Questionnaire, the Stanford Trust in Physician Scale, the Health Care Climate Questionnaire, Schmidt Perception of the Nursing Care Survey, Person‐Centred Climate Questionnaire, the Individualized Care Scale, the Patient‐Centred Inpatient Scale, Patient Satisfaction with Nursing Care Quality Questionnaire, Short Form‐36 and Functional Independence Measurement. Only two studies discussed possible empirical referents in the obstetrical care studies, which included perinatal outcomes (preterm birth, perinatal death), decisional regret scores and the Shared Decision‐Making Questionnaire (SDM‐Q‐9).

Overall, there was significant overlap in the defining attributes identified between PCC and obstetrical care with six common themes. There were five PCC and six obstetrical unique themes. Antecedents were commonly divided into patient factors, provider factors, organisational capacity, systematic factors and mutual participation and communication. Few PCC and obstetrical care studies listed empirical referents. In total, 13 PCC and 3 obstetrical empirical referents were identified across the review.

## 4 DISCUSSION

The aim of the review was to compare and contrast how care was delivered to patients between PCC and obstetrical care based on a Walker and Avant concept analysis. Common themes between PCC and obstetrical care include respect and dignity, informed decision‐making, therapeutic alliance, effective communication, social relationships and autonomy. There were unique elements to high‐quality obstetrical care including continuity of care, privacy and confidentiality, provider education and status, physical environment and equitable maternal care. Two concept analyses were conducted independently to synthesize the current literature for PCC and obstetrical care. There was significant overlap in the defining attributes, antecedents and consequences. DeLabrusse et al.^55^ had cross‐referenced PCC definitions with maternity care and found that one model^56^ was inclusive of high‐quality maternity care, indicating that there may be the applicability of some PCC models of care to high‐quality maternity care. However, there were still a significant number of themes that were unique to PCC and obstetrical care, indicating that some aspects of high‐quality intrapartum care cannot be entirely explained using a PCC framework. Many of the defining attributes identified are similar to those found in other studies about PCC and obstetrical health. For instance, the WHO quality of care framework for maternal and newborn health also included dimensions such as communication, respect and dignity and emotional support.^57^ This also aligns with the current PCC interventions to improve the quality of facility‐based delivery, which primarily pursued the PCC objectives of autonomy, supportive care, social support, the health facility environment and dignity.^58^

This review also highlighted several gaps in the understanding of PCC and obstetrical care. Few reviews included definitions of PCC and high‐quality maternity care. The several studies that did define these concepts varied significantly, indicating that there is no unifying definition for either concept yet in the literature. Indeed, despite the large body of literature on PCC, there is no consensus on a definition of PCC^59^ or maternity health.^60^ This study also revealed the lack of empirical referents for assessing obstetrical care. While there were many tools and validated scales for PCC, there were few that were identified for high‐quality obstetrical care. The consequences for high‐quality care tended to be more focused on newborn mortality, maternal mortality and health outcomes rather than quantifying the quality of maternity care. This indicates that there is a need for the development of empirical referents for the quality of obstetrical care.

There are unique elements to obstetrical care, such as continuity of care, privacy and confidentiality, provider education and status, physical environment and equitable maternal care, as identified in this study. This may be due to the unique needs of women, particularly during childbirth. Previous literature on patient‐centred care for women (PCCW) identified that women more frequently prioritized exchanging information above other domains.^61^ This is reflected in the results of this concept analysis, as informed decision‐making was disproportionately identified as a defining feature in obstetrical care studies in comparison to PCC studies. Women experience unique health challenges that cannot be approached in the same manner as other PCC interventions due to ongoing gender disparities.^62^ However, another interpretation is that prior research on perinatal care did not fully explore women's needs, experiences and outcomes using a robust PCC framework, as was used in this study, and may have missed identifying key domains of high‐quality care. Therefore, ongoing research may be needed to more fully explore obstetrical care with a PCC lens. A modified Walker and Avant concept analysis was used due to its purpose in ‘determining the likeness and unlikeness between concepts’.^30^ This was demonstrated in this concept analysis and fulfilled the aim of the study, which was to effectively identify clear similarities and differences between obstetrical care and PCC. A thorough comparison using this framework included defining features, antecedents, consequences and outcomes. By analysing or comparing high‐quality perinatal care to PCC, we identified possible gaps in the way in which obstetrical care has been studied.

Several strengths of this study include a comprehensive search of multiple databases, compliance with the reporting of reviews^63^ and appropriate application of a pre‐existing model of concept analysis.^30^ There were several limitations to this study. We did not search the grey literature, which may have excluded several articles of interest from the search. In addition, the studies that were included varied widely from high‐income countries to low‐ and middle‐income countries. The perspectives and priorities regarding maternity health differ significantly across these different contexts. For instance, hygiene and mistreatment were common themes in low‐ and middle‐income countries, but not in high‐income country studies. In addition, the focus of the obstetrical care was specifically on the perinatal period during the labour and delivery. This excluded other periods of interest, such as antenatal care or postnatal care. We also included the general delivery experience and excluded specific clinical situations, such as ectopic pregnancies and termination of pregnancy. These situations may offer another perspective in terms of high‐quality obstetrical care.

This study revealed several ideas for ongoing research. The results of this concept analysis highlight the need for more high‐quality studies evaluating the definition of high‐quality maternity care, particularly with a PCC lens. There needs to be a more standardized definition and model of care for both PCC and obstetrical care that is widely applicable. This study has shown that there is a significant intersectionality between the two concepts and that PCC models may be applicable to aspects of maternity care. A more centralized PCC model for women would need to take into consideration different settings and conditions, including obstetrical care. This study can help inform future changes to health system design, health policy and healthcare delivery.

## 5 CONCLUSION

There have been many studies that have reviewed PCC and high‐quality obstetrical care as separate entities; however, there continues to be variation in how PCC and high‐quality obstetrical care is defined. Our aim with this review was to present findings from reviews on the concept of PCC and high‐quality obstetrical care since 2010 to understand how they have been conceptualized. Furthermore, more research is needed both within PCC and obstetrical health to organize, define and categorize information related to women's healthcare. A paradigm shift in women's health as a concept is essential to deliver care that is more encompassing of the needs and priorities of women in different aspects of their health and over the course of their lifespan. With more research in care delivery for different conditions for women, information on the preferences and needs of female patients can be used to create a comprehensive and holistic framework for PCCW. This framework can then be utilized in policy and guideline development to effectively meet and address the needs of female patients, or provide a female patient perspective to existing guidelines that tend not to emphasize women's experiences.

## CONFLICT OF INTERESTS

The authors have no conflicts of interest to declare.

## Supporting information

Supporting information.

Supporting information.

Supporting information.

## Data Availability

The data that support the findings of this study are available from the corresponding author upon reasonable request.
