# Supplementary material for: How is patient‐centred care conceptualized in obstetrical health? comparison of themes from concept analyses in obstetrical health‐ and patient‐centred care
Source: Health Expect. 2022 Jan 13;25(3):823–39. doi: 10.1111/hex.13434 (PMC9122412; doi:10.1111/hex.13434)
Supplement: Supplementary file 1 — Supporting information. [file HEX-25--s002.docx]

Supplementary File 1. Inclusion and exclusion criteria based on PICO categories

| PICO Category | Include | | Exclude | | Definitions |
| --- | --- | --- | --- | --- | --- |
|  | PCC literature | Obstetrical literature | PCC literature | Obstetrical literature |  |
| Persons or participants | - Patients aged 18+ with any characteristics or conditions - Healthcare professionals of any specialty in any primary, secondary or tertiary setting of care | - Women aged 18+ - Healthcare professionals of any specialty in any primary, secondary or tertiary setting of care that provide obstetrical care to women including (but not necessarily limited to): family physicians, nurse practitioners, midwives, gynecologists, obstetricians, obstetrical internists | - Residential or long-term care settings - Palliative or end-of-life care - “patient-centred medical home”, which refers to specific primary care structures implemented in the United States - trainees | - Focus on multi- or inter-disciplinary care - trainees |  |
| Issue or intervention | Reviews that examine or describe what elements and processes constitute person-centred care | Reviews that examine or describe what elements and processes constitute high quality obstetrical care (delivery period or immediately perinatal) | - Study concludes that PCC needed but did not assess what constitutes or supports PCC - Focus on medical or clinical care | - Study concludes that higher quality obstetrical care needed but did not assess what constitutes or supports it - Focus on medical or clinical care | Define either obstetrical care in terms of the issues or procedures that are included |
| Comparisons | Reviews explore what participants view as PCC or PCC barriers, or assess if PCC is/was delivered, or evaluate PCC outcomes after an intervention, before and after an intervention, or compared between interventions | Same, but substitute high quality obstetrical care for (delivery period or immediately perinatal) | Primary research or reviews that are not of a systematic nature (can be many types but not a literature review) | Primary research or reviews that are not of a systematic nature (can be many types but not a literature review) | Reviews of a systematic nature include systematic reviews, scoping reviews, narrative reviews, qualitative reviews, realist reviews, etc. |
| Outcomes | Views, beliefs or preferences; enablers, barriers or challenges; interventions that promote or support PCC; impacts of PCC (can be patient-oriented or patient-provider, or organizational or system level | Views, beliefs or preferences; enablers, barriers or challenges; interventions that promote or support high quality care; impacts of high quality care (can be patient-oriented or patient-provider, or organizational or system level |  |  |  |
