# Supplementary material for: How is patient‐centred care conceptualized in obstetrical health? comparison of themes from concept analyses in obstetrical health‐ and patient‐centred care
Source: Health Expect. 2022 Jan 13;25(3):823–39. doi: 10.1111/hex.13434 (PMC9122412; doi:10.1111/hex.13434)
Supplement: Supplementary file 2 — Supporting information. [file HEX-25--s001.docx]

Supplementary File 2. MEDLINE search strategy

| [#](https://ovidsp-dc2-ovid-com.myaccess.library.utoronto.ca/ovid-a/ovidweb.cgi?&S=EPAOFPFIKMEBNIKNIPPJIFOGFEAGAA00&Sort+Sets=descending) | **Searches** | **Results** |
| --- | --- | --- |
| 1 | Patient-Centered Care/ | 20397 |
| 2 | (patient centered or patient-centered or patient centred or patient-centred).mp. | 38292 |
| 3 | (person centered or person-centered or person centred or person-centred).mp. | 6890 |
| 4 | (wom#n centered or wom#n-centered or wom#n centred or wom#n-centred).mp. | 639 |
| 5 | 1 or 2 or 3 or 4 | 43912 |
| 6 | Delivery, Obstetric/ | 30314 |
| 7 | Obstetrics/ | 23155 |
| 8 | "Obstetrics and Gynecology Department, Hospital"/ | 2731 |
| 9 | (obstetric* or birth or postnatal or perinatal or "labor and delivery" or "labour and delivery").mp. | 621615 |
| 10 | 6 or 7 or 8 or 9 | 621615 |
| 11 | Patient-centered care/ | 20397 |
| 12 | Patient satisfaction/ | 84037 |
| 13 | Quality assurance, health care/ | 56350 |
| 14 | Quality improvement/ | 27123 |
| 15 | Quality indicators, health care/ | 16237 |
| 16 | Quality of health care/ | 74453 |
| 17 | Program evaluation/ | 64650 |
| 18 | Physician-patient relations/ | 73744 |
| 19 | Nurse-patient relations/ | 35617 |
| 20 | (health care quality or health care assessment or health care improvement).mp. | 3743 |
| 21 | 11 or 12 or 13 or 14 or 15 or 16 or 17 or 18 or 19 or 20 | 407389 |
| 22 | 10 and 21 | 12746 |
| 23 | 5 or 22 | 53254 |
| 24 | limit 23 to (english language and yr="2010 -Current" and "all adult (19 plus years)") | 13234 |
| 25 | limit 24 to ("review" or "systematic review") | 755 |
| 26 | (concept analysis or critical review or scoping review or theoretical review or narrative review or meta-narrative review).ti. | 23178 |
| 27 | (concept analysis or critical review or scoping review or theoretical review or narrative review or meta-narrative review).ab. | 26726 |
| 28 | 26 or 27 | 40542 |
| 29 | 24 and 28 | 67 |
| 30 | 25 or 29 | 769 |
