# Supplementary material for: How is patient‐centred care conceptualized in obstetrical health? comparison of themes from concept analyses in obstetrical health‐ and patient‐centred care
Source: Health Expect. 2022 Jan 13;25(3):823–39. doi: 10.1111/hex.13434 (PMC9122412; doi:10.1111/hex.13434)
Supplement: Supplementary file 3 — Supporting information. [file HEX-25--s003.docx]

Supplementary File 3. Characterization of Defining Attributes and Antecedents

**Definitions of Defining Attributes**

- **Respect and Dignity:** patient choices and perspectives are respected and affirmed, freedom from harm and mistreatment
- **Informed Decision-Making:** decisions made with the patient understanding and appreciation of care options and consequences with effective information provision from the provider and patient engagement
- **Therapeutic Alliance:** building a constructive patient-provider relationship
- **Effective Communication:** two-way interaction of the provider and patient where information is conveyed and shared
- **Social Relationships:** consideration of the impact of illness and care with the patient’s family
- **Autonomy:** the patient being in ownership and having power over their own health care decisions
- **Holistic Care:** acknowledging the person’s whole life through a biopsychosocial perspective
- **Empowerment:** acknowledging the patient’s ability to self-manage and encouraging the patient to take responsibility for their own health
- **Individualized Care:** care that is tailored to the unique needs, values, and beliefs of the patient
- **Coordinated Care:** integration of different aspects of patient care
- **Empathy:** compassion, emotional support, and understanding of the patient’s perspective
- **Continuity of Care:** continuous presence of healthcare provider throughout the care experience
- **Privacy & Confidentiality:** having care and support that is private and limited to the immediate health care team
- **Provider Education & Status:** provider perceptions of their own status and authoritative knowledge and health provider education
- **Physical Environment:** hygienic facilities that allow privacy and comfort for the patient
- **Equitable Maternal Care:** availability of services for all pregnant women regardless of race, religion, ethnicity or cultural background

**Identified Antecedents**

|  | **PCC** | **Obstetrical Care** |
| --- | --- | --- |
| **Patient Factors** | - - Capacity to engage in decision-making | - - Choice predisposition, feelings   - Comfort in expressing preferences   - Sufficient information provided   - Previous experiences of labour   - Family/partner involvement |
| **Provider Qualities** | - - Vision and commitment   - Leadership   - Personal qualities of staff   - Interdisciplinary teamwork   - Knowledge and ability | - - Skilled, competent, motivated |
| **Organizational Capacity** | - - Physical environment   - Feedback and organizational learning   - Access to resources and time   - Culture that respects values and choices | - - Care guidelines and evaluation   - Availability of resources   - Health infrastructure and quality of services   - Health financing   - Continuum of care   - Location: hygienic, privacy |
| **Systemic Factors** | - - Health disparities (-)   - Access to care | - - Evidence-based care   - Communication systems   - Social stability   - Cultural sensitivity |
| **Other unique themes** | - - Mutual Participation and Communication   - Need for healthcare intervention   - Shared governance | - - Mutual Participation and Communication |
